# Drug Resistance in Non-Small Cell Lung Cancer: A Potential for NOTCH Targeting?

**DOI:** 10.3389/fonc.2018.00267

**Published:** 2018-07-24

**Authors:** Venus Sosa Iglesias, Lorena Giuranno, Ludwig J. Dubois, Jan Theys, Marc Vooijs

**Affiliations:** Department of Radiation Oncology, GROW, School for Oncology and Developmental Biology, Maastricht University Medical Center (MUMC), Maastricht, Netherlands

**Keywords:** non-small cell lung cancer, treatment resistance, NOTCH/gamma-secretase inhibitor, chemotherapy, targeted therapy

## Abstract

Drug resistance is a major cause for therapeutic failure in non-small cell lung cancer (NSCLC) leading to tumor recurrence and disease progression. Cell intrinsic mechanisms of resistance include changes in the expression of drug transporters, activation of pro-survival, and anti-apoptotic pathways, as well as non-intrinsic influences of the tumor microenvironment. It has become evident that tumors are composed of a heterogeneous population of cells with different genetic, epigenetic, and phenotypic characteristics that result in diverse responses to therapy, and underlies the emergence of resistant clones. This tumor heterogeneity is driven by subpopulations of tumor cells termed cancer stem cells (CSCs) that have tumor-initiating capabilities, are highly self-renewing, and retain the ability for multi-lineage differentiation. CSCs have been identified in NSCLC and have been associated with chemo- and radiotherapy resistance. Stem cell pathways are frequently deregulated in cancer and are implicated in recurrence after treatment. Here, we focus on the NOTCH signaling pathway, which has a role in stem cell maintenance in non-squamous non-small lung cancer, and we critically assess the potential for targeting the NOTCH pathway to overcome resistance to chemotherapeutic and targeted agents using both preclinical and clinical evidence.

## Lung Cancer and Standard of Care

According to the World Health Organization, in 2015, every 3.5 s a person died of cancer and one out of five deaths was due to lung cancer. Lung cancer is the second most commonly diagnosed type of cancer and the leading cause of cancer-related mortality. More than two thirds of lung cancer patients are diagnosed at an advance stage (III–IV). The lack of early diagnostic techniques and the intrinsic and/or acquired treatment resistance leading to relapse are major obstacles in finding a cure for lung cancer.

Lung cancer can be divided into two main categories: non-small cell lung cancer (NSCLC) accounting for 85% of lung cancers, and small cell lung cancer (~15%). NSCLC can be further categorized into, generally, adenocarcinoma (AC 40%), squamous cell carcinoma (SQCC 25–30%), large cell undifferentiated carcinoma (10–15%), mixed subtypes (adenosquamous), and the far less common sarcomatoid carcinoma. Treatment for NSCLC consists of surgical resection, chemotherapy, radiation, targeted therapy, immune therapy, and/or combinations thereof. Standard first-line treatment for inoperable locally advanced stage NSCLC is concurrent polychemotherapy with fractionated radiation (60 Gy in 2 Gy fractions) (1). Studies show that chemoradiotherapy (using paclitaxel) as opposed to radiotherapy alone, delivered after induction chemotherapy (carboplatin and paclitaxel), is feasible and improves time to progression and overall survival (OS) of inoperable stage III NSCLC (2).

Polychemotherapy for NSCLC often involves the combination of a platinum-based agent (e.g., cisplatin or carboplatin) and other drugs with a different mechanism of action. Cisplatin or carboplatin covalently binds DNA, activates the DNA-damage response, and induces cell cycle arrest and apoptosis. The second chemotherapeutic agent can be a topoisomerase II inhibitor (e.g., etoposide), a DNA damaging agent preventing replication such as a taxane (e.g., paclitaxel or docetaxel) or a vinca alkaloid (e.g., vincristine, vinorelbine, or vinblastine) which inhibits microtubule assembly and blocks mitosis, an altered DNA base that gets incorporated in the DNA but cannot be repaired (e.g., gemcitabine), or an inhibitor of folate metabolism (e.g., pemetrexed). There are studies that suggest that the selection of the chemotherapeutic agent should consider the subtype of NSCLC. Second-line paclitaxel treatment for cisplatin-treated lung cancer patients benefits clinical outcome (response rate plus stable disease) in non-squamous cell carcinomas preferentially (3). Adenocarcinoma seems to have better OS rates for both gemcitabine-platinum and taxane-platinum regimens, where the first, results in better objective response rates and shows a tendency to improve median survival time (9.1 versus 7.4 months in the taxane combination) (4). Squamous cell carcinoma patients could benefit more from a cisplatin plus etoposide treatment rather than the four-drug combination: cyclophosphamide, adriamycin, methotrexate, and procarbazine, where the response rate is 44.7 versus 21.6%, respectively (5). Large cell neuroendocrine carcinomas and small cell lung carcinomas have a similar biological behavior and respond similarly to some treatments including: irinotecan, platinum, and taxanes, which are more effective than pemetrexed (6–8). The remaining subtypes of large cell carcinomas (non-neuroendocrine), sarcomatoid tumors, and mixed carcinomas often do not have well-defined biological features, the criteria for diagnosis are not as robust, and hence, treatment response has not been properly assessed. Regarding targeted agents, several studies suggest that adenocarcinoma patients benefit more from epidermal growth factor receptor (EGFR)–tyrosine kinase inhibitor (TKI) therapy than squamous cell carcinoma patients, both subtypes bearing *EGFR* mutations, where objective response rates, OS, and progression-free survival (PFS) are 66–74%, 19–21 months, and 9.4–10 months (9) versus 25–27%, 13.48 months, and 3–5 months (10, 11), respectively. Within the adenocarcinoma subtype, the brochioloalveolar one is the most responsive to small molecule tyrosine kinase inhibitors (TKI) (e.g., gefitinib) (12). These observations raise the following question: “which are the reasons behind these diverse responses and outcomes to the same treatments between lung cancer subtypes and patients?”

## The Lung Cancer Genome: Actionable Targets in NSCLC?

Whole genome sequencing of lung cancers has revealed complex patterns of driver mutations with over 200 non-synonymous mutations that distinguish smokers from non-smokers and predict patient outcome (13–15). Mutations in *KRAS* occur in up to 25% of NSCLC and despite preclinical efforts, there are no clinically approved drugs that effectively target KRAS. In lung adenocarcinoma, actionable mutations in the epidermal growth factor receptor (*EGFR*) occur with a 10–15% frequency and can be effectively targeted with small molecule first- and second-generation tyrosine kinase inhibitors (TKI) (e.g., erlotinib, gefitinib, and afatinib) and monoclonal antibodies (mAbs) (e.g., cetuximab). TKIs that target translocations in the anaplastic lymphoma kinase (e.g., EML4-ALK) occurring with a 5% frequency in adenocarcinomas, are also available (e.g., ceritinib, alectinib, and crizotinib). Other actionable driver mutations (~15–20%) that occur less commonly are *ROS1* rearrangements, *BRAF* mutations, *RET* rearrangements, *NTRK1* rearrangements, *MET* amplifications, and *HER2* mutations. In about 40% of lung adenocarcinomas however, there are no common driver genes yet identified (16). High response rates (60–70%) are achieved with the EGFR TKIs in *EGFR*-mutated cancers (9) and ~60% of partial/complete responses with anaplastic lymphoma kinase (ALK) inhibitors (e.g., crizotinib) in patients with *ALK* translocations (17). However, resistance to pharmacological inhibitors, for example, TKIs, seems inevitable. Mechanisms of resistance include: alteration of the drug target such as resistance mutations, alternative splicing, and gene amplification, as well as activation of alternative oncogenic pathways. Tumor cells which harbor these resistance-creating mutations can be present at the onset of treatment (primary resistance) or emerge during treatment (secondary resistance). Other mechanisms of resistance, for instance inefficient drug delivery, metabolic inactivation and drug-interactions, also play a role in therapeutic outcome. The most frequent form of acquired resistance in NSCLC is secondary mutations in *EGFR* (e.g., T790M “gatekeeper”) occurring in 60% of patients treated with second generation TKIs. Similarly, secondary mutations in *ALK* (e.g., C1156Y, L1196M, G1269A, and L1152R) are associated with acquired resistance to first generation ALK inhibitors such as crizotinib. In addition, there are several pathways that can mediate resistance to TKI which include the activation of anti-apoptotic pathways, *HER2* and *MET* amplification, or mutations in *PIK3CA* or *BRAF* (18).

In the squamous cell carcinoma subtype of non-small cell lung cancers (SQCC NSCLC), most tumors carry mutations in *TP53, RB1*, and *CDKN2A* and in the oxidative pathway genes and *NFE2L2*. *EGFR* and *ALK* mutations, common in adenocarcinomas, are less frequent in SQCC of the lung and hence, agents developed for lung adenocarcinoma are less effective against lung SQCC. In adenocarcinoma patients, EGFR–TKI objective response rates, OS, and PFS are 66–74%, 19–21 months, and 9.4–10 months (9) versus 25–27%, 13.48 months, and 3–5 months for SQCC (10, 11), respectively. Interestingly, SQCC differentiation genes such as *SOX2* and *TP63* (*TP53* homolog) are commonly altered and mutually exclusive with loss-of-function mutations in *NOTCH1* and *NOTCH2*. Other alterations include amplification of EGFR, FGFR1, and PI3K pathways (19). Alternative approaches that target the tumor microenvironment using anti-angiogenic therapies such as antibodies or small molecule inhibitors aimed at the vascular endothelial growth factor (VEGF) or its receptor (VEGFR), were the first targeted agents to yield a significant improvement in OS when combined with first-line chemotherapy for metastatic NSCLC. Anti-angiogenic treatment however, also resulted in strong normal tissue toxicities (20). Importantly, anti-angiogenesis inhibition combined with platinum chemotherapy does not improve outcome for squamous NSCLC (21).

Remarkable PFS rates have been observed in advanced NSCLC using immune checkpoint inhibitors (e.g., nivolumab and ipilimumab) as first-line treatment, superior to chemotherapy in both squamous and non-squamous NSCLC (22). Checkpoint inhibitors that target PD-L1/PD-1 and CTL-A4 receptors expressed on immune and tumor cells, block the antitumor adaptive immune response by suppressing the cytotoxic T-cell response. mAbs that block the interaction between PD-L1 and PD-1 (e.g., durvalumab) improve PFS [16.8 versus 5.6 months (placebo)], the response rate, and its duration (72.8 versus 46.8% 18-month response) in stage III unresectable NSCLC pre-treated with platinum-based chemotherapy (23, 24). There are still many factors that remain uncertain that would enable clinicians to determine the response to checkpoint inhibitors but a high mutation load creates immunogenic tumors and is strongly associated with response to checkpoint inhibitors (25). Unfortunately, most NSCLC patients do not respond to such immunotherapies despite expressing PD-L1, and the disease progresses, indicative of resistance to checkpoint inhibitors (26).

Better and more holistic approaches have been proposed showing that a “cancer mutation signature” is more predictive for treatment response than the individual mutation status (27). In *KRAS*-driven NSCLC, the signature—*FOXRED2, KRAS, TOP1, PEX3*, and *ABL2*—was more predictive for prognosis than the single mutation status of *KRAS* (28). An RNA-sequence-based prognostic model built with four genes (*RHOV, CD109, FRRS1*, and *LINC00941*) was statistically associated with worse OS and metastasis-free survival, and is able to stratify patients bearing *KRAS* or *EGFR* mutations versus their wild-type counterparts in OS outcome (29). Because lung cancer is a highly heterogeneous disease on the genetic, epigenetic and metabolic levels, it is perhaps not so surprising that personalized medical approaches targeting only one driver mutation improves OS but cannot increase cure rates.

## Lung Cancer Heterogeneity

Cancers are composed of mixed cell populations with diverse genotypic, epigenetic, phenotypic, and morphological characteristics. Tumor heterogeneity is observed among different patients with the same tumor subtype (interpatient heterogeneity), among tumor cells within one host organ (intratumor heterogeneity), between the primary and the metastatic tumors (intermetastatic heterogeneity), and among tumor cells within the metastatic site (intrametastatic heterogeneity) (30). It was first exemplified in renal cancer that biopsies from primary and metastatic sites from the same patient showed extensive divergent and convergent evolution of driver mutations, copy number variations, and chromosome aneuploidy (31). It has been proposed for a long time now that these subclonal tumor populations, present at low frequency, contain clones with invasive and metastatic properties (32), and are able to escape the effect of systemic and targeted treatments, thus affecting clinical outcome. It is well understood that heterogeneity is not only determined by cell intrinsic mechanisms but also by the dynamic tumor microenvironment (e.g., angiogenesis, immune system, fibroblasts) (33). Lung cancer is also highly heterogeneous with respect to metabolic activity and blood perfusion at the macro-level as well as at the single-cell level (34, 35). Genome sequencing in NSCLC has identified hundreds of mutations present in subclonal fractions that increase with tumor-grade (13, 36), and in primary tumors, predict early postsurgical relapse (37). Smokers have 10-fold more mutations than non-smokers and distinct driver mutations (e.g., *EGFR* versus *KRAS*) (13, 14). Chromosomal instability, which is a driver of intratumor heterogeneity, is associated with anti-cancer drug resistance, and is associated with poor outcome in NSCLC. Tumor subclones have different actionable therapeutic targets explaining the variety of responses to targeted therapeutics (38, 39). In addition to the genetic and epigenetic heterogeneity, there is a high degree of heterogeneity in tumor metabolism which is highly dynamic and subject to changes in oxygen, nutrients and other tumor microenvironmental factors (35). Taken together, the different levels of heterogeneity in tumors are of high clinical relevance in tumor progression, treatment response, and relapse. One of the main genetic drivers of tumor heterogeneity are cancer stem cells which create and maintain a tumor cell hierarchy (40).

## Lung CSCs

Cancer stem cells were first identified in myeloid leukemias by Dick and colleagues. CSCs are tumor-initiating cells responsible for the cellular hierarchy maintained by means of self-renewal, and causing tumor heterogeneity, and are capable of multipotent differentiation (41). Tumor heterogeneity may also be due to the plasticity of CSCs which enables them to differentiate reversibly into different cell types under specific environmental conditions (42). Furthermore, differentiated cancer cells may be reprogrammed to a more stem cell-like state under specific conditions (e.g., hypoxia induces OCT4 and NANOG) (43) and hence, contribute to recurrence. In addition, chromosomal instability together with external environmental factors, may lead to CSC heterogeneity and even to metastasis.

Cancer stem cells from NSCLC have the ability to form colonies in soft-agar, they are highly tumorigenic *in vivo* (44), and can be identified by virtue of Hoechst dye efflux (the side population, SP) using flow cytometric methods. CSCs express multidrug ATP-binding cassette (ABC)-transporters and are resistant to multiple chemotherapeutic agents (45). One of the best characterized CSC markers for solid cancer, including NSCLC, is the CD133 cell surface protein. CD133-expressing (CD133+) lung cancer cells are self-renewing tumor cells that express markers from embryonic stem cells, are present in low numbers in human NSCLC, but are highly tumorigenic. Moreover, when CD133+ CSC differentiate, their CD133− progeny is no longer tumorigenic (46). It seems plausible that combination therapy targeting dually and specifically stem cells and non-stem cells would be required to be successful in, or at least be closer to, eradicating cancer (47). There is now mounting evidence that the normal stem cell pathways such as WNT, NOTCH, and HH (Hedgehog) are deregulated and mutated in cancer and CSCs (48). NOTCH signaling plays a role in the maintenance of CSCs in different cancer types including T-cell acute lymphoblastic leukemia (T-ALL) (49), brain (50), breast (51), colon (52), and lung cancer (53).

## The Canonical NOTCH Signaling Pathway

The NOTCH signaling pathway is a highly conserved cell-to-cell communication pathway between cells expressing the single pass transmembrane NOTCH receptor and neighboring cells expressing a transmembrane NOTCH ligand. It is a major cell fate determination pathway essential for embryonic development. In adult tissues, NOTCH signaling regulates tissue homeostasis through cell renewal, differentiation, proliferation, and cell death (54, 55). The mammalian genome encodes for four NOTCH receptor genes (*NOTCH* 1–4) and five NOTCH ligands (*JAGGED1, 2* or *DELTA1, 3*, and *4*). NOTCH signaling begins at the cell surface and is highly regulated by the proteolytic cleavage of the NOTCH receptor. NOTCH receptors are transported to the cell surface as furin-cleaved heterodimers and ligand interaction initiates two consecutive proteolytic cleavages. The first proteolytic cleavage is executed by the ADAM10 metalloprotease, which cleaves the NOTCH ectodomain, and is followed by the intramembranous and rate-limiting cleavage by the γ-secretase complex (56). The γ-secretase liberates the NOTCH intracellular domain (NICD) from the cell membrane, and is then translocated to the nucleus where it binds to the DNA-bound protein CSL (also called RBP-Jk), and together with the Mastermind (MAML) co-activators, forms the NOTCH transcriptional complex. In the C-terminal end of the NICD, there is a proline/glutamic acid/serine/threonine-rich motif (PEST) which is a substrate for the E3 ubiquitin ligase FBWX7, and targets NICD for proteasomal degradation when the signal needs to be shut down.

NOTCH regulates the transcription of genes of the *HES* and *HEY* family, *CD25* and *GATA3* (in T cells), negative regulators of NOTCH signaling (e.g., *NRARP, DELTEX1*), oncogenes like *RAS, CYCLIN D1, P21/WAF1*, and *C-MYC*, among many others (57). NOTCH signaling has been found deregulated in multiple human diseases, and recently, there is growing evidence supporting the role of NOTCH signaling in the development and progression of cancers (58). Gain-of-function mutations are a hallmark in T-ALL (59), but overexpression and mutations in NOTCH receptors members are found at lower frequencies in many other leukemias and solid cancers. A recent review summarized the involvement of NOTCH signaling in all acquired capabilities of cancer cells, already defined by Hanahan and Weinberg as the hallmarks of cancer (60, 61).

## NOTCH in a Physiological and Pathological Context in the Lung

NOTCH receptors and ligands are expressed during early lung development and control cell fate specification and branching along the proximal-distal axis (62, 63). NOTCH blockade reduces the number of SOX2 progenitors and alters the balance between basal, ciliated, neuroendocrine, and secretory cell fates in the airway epithelium (64, 65). In the postnatal lung, NOTCH restricts basal cells to the secretory cell fate suppressing ciliated differentiation (66). NOTCH signaling is also required to maintain the differentiated state of secretory cells in the upper airways and blocking NOTCH-JAGGED1/2 signaling leading to transdifferentiation of club cells into ciliated cells (67). NOTCH2 regulates differentiation of lineage-restricted progenitors into bronchial club cells and ciliated cells as well as contributing to alveolar morphogenesis and integrity of epithelial and smooth muscle layers of airways (68, 69). NOTCH1/3 contribute additively to regulate pulmonary neuroendocrine cell fate (68). While NOTCH1 is dispensable for airway epithelial development upon lung epithelial injury, NOTCH1 is essential to induce club cell regeneration by activating its downstream targets HES5 and PAX6 (70).

Deregulation or mutation of NOTCH receptors, ligands, and signaling regulators is associated with pathogenesis of many hematological and solid tumors including lung cancer (60). In T-cell leukemias, *NOTCH1* activating mutations occur in 60% of cases. Many human lung cancer cell lines (20%), and primary lung cancers, harbor missense or non-sense mutations in one of the NOTCH receptors (71). Translocations involving *NOTCH3* were first identified in NSCLC (72), are found overexpressed in 30% of NSCLC, and are strongly correlated with EGFR expression (73). Gain-of-function mutations in *NOTCH1* or loss of the negative NOTCH regulator *NUMB* have been identified in up to 30% of adenocarcinomas and are correlated with poor prognosis. Loss of *NUMB* is also correlated with higher NOTCH activity, and in tumors with wild-type *TP53*, NOTCH1 expression was associated with worse outcome (74). Furthermore, high expression of NOTCH1 and NOTCH3 receptors, ligands, and target genes is correlated with worse survival in resected NSCLC (53, 75, 76). Other studies have shown conflicting data on the role of NOTCH1 expression and its influence on the outcome in NSCLC (77, 78). It must be noted, however, that lung cancers were not sub-classified into adenocarcinoma and squamous carcinoma in all studies. A meta-analysis confirmed positive correlations of NOTCH1 and 3 expressions with progression and worse OS in adenocarcinoma (but not in lung SQCC) (79). For the NOTCH ligand DLL3 and the target gene *HES1*, significant associations between expression and worse OS have been found in adenocarcinoma (53, 76, 79). In addition to its oncogenic role, inactivating mutations in NOTCH receptors has also been associated with squamous cancers of the skin, head and neck, and lung (80). The tumor suppressive role of NOTCH in epidermal differentiation was first identified in mice with keratinocyte-specific loss of *Notch1* which developed skin carcinoma (81). More recently, sequencing analysis has identified missense and non-sense mutations in SQCC in *NOTCH1* or *2* that suggest a loss of function (82, 83), but no loss-of-function mutations have been reported for NOTCH ligands or target genes.

## Oncogene Addiction and Treatment Resistance

In 2002, Weinstein proposed a potential Achilles heel of cancer which he referred to as *oncogene addiction*, whereby the expression of oncogenes is not only required for the initiation of tumorigenesis but also for the maintenance of the malignant phenotype (84). This concept was coined based on findings from preclinical studies in which tumors regressed when *C-MYC, KRAS, TP53*, and other commonly mutated oncogenes that were used to initiate the tumors, were turned off. Unfortunately, two decades later, there are still no clinically approved therapeutics against MYC, RAS, or TP53. To date, the only pharmacological proof of oncogene addiction in patients is in chronic myelogenous leukemia were tumors regress and are cured upon targeting the *BCR-ABL* fusion gene with the small molecule inhibitor serine/threonine kinase inhibitor gleevec. To survive, tumor cells evolve by either promoting the emergence of new tumor clones that are no longer dependent on the initial activating oncogene (primary resistance), or by developing mutations (of the drug target or downstream activating mutations) that make tumors that were initially responders, insensitive to monotherapy treatment (secondary resistance). For example, during anti-EGFR treatment (e.g., cetuximab) of metastatic colorectal cancer, *KRAS*-mutant cells can be identified in the blood of patients while tumors are still regressing (85). Despite the paramount clinical success of gleevec, resistance also develops by acquisition of mutations in the binding site of gleevec (86). It is evident that monotherapies of the currently-used targeted agents will not lead to cancer cure, therefore, combination of therapies is required. Given the important role of NOTCH signaling in CSCs and its frequent involvement in NSCLC, we asked ourselves whether NOTCH-based therapy combined with systemic chemotherapy or targeted agents was a promising path to pursue.

## NOTCH-Related Resistance to Chemotherapy

### Platinum-Based Drugs (Cisplatin, Carboplatin)

Platinum-based drugs bind covalently to DNA thereby interfering with replication, particularly in fast-growing cells, and prompt activation of DNA-damage recognition and repair mechanisms leading to cell cycle arrest or apoptosis when repair is not effective. *In vitro* and *in vivo* studies show that cisplatin enriches a subpopulation of NOTCH-regulated CD133-expressing stem-like lung cancer cells that cause cross-resistance to paclitaxel and doxorubicin by upregulation of ABC drug transporters: ABCG2 and ABCB1 (also called MDR-1 or P-glycoprotein) (87). Besides CD133 expression, NSCLC cells expressing CD44, NANOG, OCT4, SOX2, and ALDHA1 were shown to be resistant to cisplatin through NOTCH3-mediated activation of autophagy (88). Lung CSCs downregulate *AQP2* and *CTR1* drug transporter genes, consequently leading to reduced drug uptake and intracellular accumulation, increased DNA damage, and resistance to treatment. Moreover, these cells display an increased ability to repair cisplatin-induced DNA intrastrand cross-links *via* activation of nucleotide-excision and mismatch repair pathways (89).

Cisplatin-resistant stem-like cells also display upregulated epithelial-to-mesenchymal transition (EMT) markers (90). EMT is physiologically important during embryogenesis and it involves the loss of cell-to-cell junctions, loss of epithelial (e.g., E-cadherin/CDH1) and gain of mesenchymal markers (e.g., N-cadherin and Vimentin). Pathologically, it is involved in tumorigenesis, metastasis and therapeutic resistance. NOTCH induces EMT *via* activation of transcription factors including TWIST, SNAIL, SLUG, and ZEB (91). In addition, NOTCH expression has been shown to be regulated by certain growth factors involved in EMT including fibroblast growth factor (FGF) and platelet-derived growth factor (PDGF) (92).

Poor prognosis of NSCLC patients with activated NOTCH signaling (either by NOTCH receptor activating mutations or loss of NUMB repressor) has been associated with absence of mutations in the tumor suppressor protein TP53 (74). Aberrantly high TP53 expression before the start of treatment is associated with strong histopathological responses (e.g., necrosis and fibrosis) to cisplatin, and it has been reported that in only 13% of the cases, there is an alteration of TP53 expression levels before and after cisplatin treatment in stage IIIA NSCLC (93). Interestingly, NUMB, a suppressor of NOTCH, forms a tri-complex with TP53 and its ubiquitin ligase HDM2 to prevent ubiquitination and consequent TP53 degradation (94). Therefore, in cancers with loss of NUMB, such as some breast cancers and lung adenocarcinomas, there is an increase in NOTCH receptor and a decrease in TP53 protein expression levels thus enhancing chemoresistance. In addition, CSL/RBP-Jĸ, a DNA binding protein that mediates NOTCH transcriptional activation, can be negatively regulated by TP53 which in turn can decrease CSL expression as feedback inhibition (95). There is evidence that NOTCH3 signaling in ovarian cancer is also predictive for platinum resistance (96). NOTCH3 signaling is active in drug-resistant CSCs and NOTCH3 inhibition induces chemosensitivity to platinum-based drugs. The preclinical and clinical data here discussed suggests that not only platinum-sensitive but also platinum-resistant cancers may benefit from NOTCH targeting. However, whether NOTCH targeting induces platinum sensitivity or not, in NSCLC patients, is not known.

### Microtubule-Targeting Inhibitors (Taxanes, Vinca Alkaloids)

Taxanes (docetaxel and paclitaxel) and vinca alkaloids (vinblastine, vincristine, and vinorelbine) interfere with microtubule function by preventing either depolymerization (taxanes) or microtubule formation (vinca alkaloids), and ultimately blocking cell cycle progression through mitosis. Overexpression of ABC drug transporters (ABCB1/MDR-1/P-gp) mediates resistance toward taxanes and vinca alkaloids, and is a common feature of human cancer including NSCLC (97). The microRNA miR-451 is a direct regulator of the multidrug resistance 1 (MDR-1) protein. Overexpression of miR-451 induces chemosensitivity while miR-451 loss induces taxane resistance in NSCLC. NOTCH1, through the activation of AP1, an early transcription factor necessary for progression through G1 phase, downregulates miR-451. NOTCH blockade using gamma-secretase inhibitors (GSIs) increases miR-451 and reduces MDR-1 thereby sensitizing tumors to taxane-based treatment (98). In docetaxel-resistant lung cancer cell lines, miR-451 is downregulated. This is turn, causes MYC/ERK-dependent inactivation of glycogen synthase kinase 3 (GSK-3β), Snail activation, and EMT (99). Whether the EMT-induced docetaxel resistance in these models is reversible or not, by blocking NOTCH, is not yet known. In a recent study, a small molecule γ-secretase inhibitor, BMS-906024, sensitized NSCLC cell lines to paclitaxel, and both drugs synergized preclinically by targeting the paclitaxel-induced increase in NOTCH1, especially in cell lines with a *KRAS* and *BRAF* wild-type background versus their mutant counterparts, in a TP53-dependent manner (100).

Factors from the immune system, the stroma, and from cancer cells, secreted by paclitaxel-resistant lung adenocarcinoma cells, contribute to acquire drug resistance by promoting cell proliferation and escaping apoptosis. However, the secretion of some of these cell-growth promoting factors is reduced when glucose is deprived. It has been shown that FOXO3a promotes cross-resistance (e.g., to 5-fluorouracil and cisplatin) *via* glycolysis-mediated ABCB1 upregulation. Suppression of the cellular energy supply by targeting glycolysis may alternatively overcome acquired drug resistance (101). Genes encoding proteins involved in glucose uptake, glycolysis, lactate to pyruvate conversion, and repression of the tricarboxylic acid cycle are direct transcriptional targets of NOTCH signaling. NOTCH upregulation in breast cancer cells leads to increased glycolysis through activation of the PI3K/AKT pathway, whereas endogenous NOTCH signaling decreases mitochondrial activity and induces glycolysis in a TP53-dependent manner (102).

In addition, NOTCH signaling also cross-talks with HIF-1α, an important glycolysis regulator (103), through physical interaction with N1 ICD under hypoxia, upregulating NOTCH downstream targets (*HES1, HEY2* mRNA) and preventing differentiation in cortical neural stem cells thus maintaining stemness (104, 105). Hypoxia (≤2% O_2_) can induce multidrug resistance (e.g., to cisplatin, carboplatin, paclitaxel, and gemcitabine) in NSCLC *via* upregulation of ABCB1 and EGF-like domain 7, an endothelial secreted factor that regulates vascular tube formation (89). Microtubule-targeting agents shift the binding of HIF-1α from actively translating polysomes to inactive ribosomal subunits as for *HIF-1*α mRNA translation requires active transport on interphase microtubules (106). HIF-1α can also be upregulated and stabilized in an oxygen-independent manner by oncogene signaling through the PI3K/AKT and MAPK/ERK/RAS pathways; both of which are found mutated in human NSCLC. NOTCH1 activates AKT-1 *via* PTEN repression and induction of the insulin-like growth factor 1 receptor (IGF-1R) in lung adenocarcinoma during hypoxia (107). The interaction between the NOTCH and hypoxia/HIF pathways thus connects two cancer vulnerabilities. Therefore, changes in the tumor microenvironment that alter energy metabolism, or requirements of tumor cells, could be exploited as targets to increase drug sensitivity using NOTCH-based therapies.

### Etoposide

Topoisomerase II enzymes are important in DNA unwinding, strand excision, and re-ligation during replication, and cell cycle checkpoint activation after DNA damage. Etoposide is a topoisomerase II inhibitor and induces cell cycle arrest and apoptosis. Etoposide resistance in NSCLC has been partly attributed to NF-κB-mediated ABCB1 drug transporter expression (108). Upregulation of NF-κB signaling, through loss of *TP53* and *KRAS* mutations, is found in different cancers including lung adenocarcinoma (109). NF-κB has been shown to function downstream of NOTCH and facilitates NOTCH target gene expression and tumor formation in pancreatic and T-ALL models (110–112). Conversely, in breast CSCs, NF-κB upregulates JAGGED1 expression and activates NOTCH signaling (113). Also, in glioblastoma multiforme, NF-κB/STAT3 signaling pathway regulates the activation of the NOTCH pathway (114). Etoposide resistance may occur *via* the 5′-tyrosyl DNA phosphodiesterase (TDP2), a transcriptional target of mutant *TP53* that repairs topoisomerase-mediated DNA damage (115). As opposed to normal lung patient tissue, 58.5% of cancer tissues that stained positive for TP53 were also positive for TDP2 (116). Although no direct link between TDP2 and NOTCH has yet been found in NSCLC, NUMB might have a role based on its involvement in TP53 degradation (94).

### Pemetrexed

Pemetrexed inhibits thymidylate synthase, dihydrofolate reductase, and glycinamide ribonucleotide formyltransferase, enzymes involved in folate metabolism, purine and pyrimidine synthesis necessary for DNA and RNA synthesis. Pemetrexed and cisplatin are often administered concomitantly and have good OS outcomes in non-squamous NSCLC as first-line therapy (21, 117) but treatment resistance is common. Pemetrexed treatment induces replicative stress in the form of single strand breaks, that if they are not repaired, will lead to the formation of double strand breaks. Cisplatin on the other hand, induces mainly intrastrand DNA cross-links which need nucleotide-excision repair pathways to be repaired, dependent on the availability of a great number of nucleotides. Pre-treatment with pemetrexed with sequential cisplatin administration results in additive/synergistic effects in NSCLC cells (118, 119). Interestingly, the surviving clones to sequential pemetrexed-cisplatin treatment frequently undergo EMT conversion and are enriched for CSCs (*CD133, CD44, NANOG*, and *OCT4B* mRNA upregulation). Blocking EMT with a natural flavonoid, such as kaempferol, overcomes resistance to anti-folate therapy in NSCLC (120). Based on its role in EMT induction and CSC maintenance, targeting NOTCH signaling in NSCLC might be an interesting option to overcome pemetrexed resistance.

### Gemcitabine

Gemcitabine is an anti-metabolite analog of deoxycytidine that is incorporated into DNA and blocks DNA replication. Gemcitabine treatment in lung cancer cell lines induces an increase in Beclin-1-mediated autophagy activation (121, 122). Inhibition of autophagosome formation using 3-methyladenine, an inhibitor of PI3K, in gemcitabine-resistant lung cancer cells, increases the expression of apoptotic mediators (121, 122). NOTCH3 is upregulated in patients with gemcitabine resistance and its knock-down reduces autophagy (LC3-II expression), colony and sphere forming ability in lung cancer cell lines (88). Since NOTCH signaling also regulates PI3K/Akt signaling, NOTCH inhibition could lead to similar effects as those obtained with 3-methyladenine. Both in lung and pancreatic tumor models, NOTCH inhibition with GSI or mAbs targeting NOTCH2/3 (e.g., tarextumab) sensitizes tumors to gemcitabine (123, 124).

## NOTCH-Related Resistance to Targeted Therapies

### EGFR Inhibitors

Epidermal growth factor receptor mutations occur in ~10–25% of lung adenocarcinoma patients and these patients benefit, at least initially, from neutralizing treatment with mAbs (e.g., cetuximab and necitumumab) and from TKI (e.g., erlotinib, gefitinib, and afatinib) (16). Often, resistance to TKI occurs *via* mutation of *EGFR* (e.g., T790M or S492R), oncogenic shift (*MET* amplification, *HER2* upregulation, and *KRAS* activation), impairment of apoptosis (e.g., BH3 domain deletions of *BIM*), and EMT conversion (125). Some patients could regain sensitivity to TKI after discontinuing TKI treatment (126). The *EGFR* T790M mutation occurs in 62% of the patients with acquired resistance and has fueled the development of second and third generation TKI (e.g., Osimertinib/AZD9291) which was FDA-approved for *EGFR* T790M positive NSCLC patients (127). However, resistance against AZD9291 has already been described *via* an alternative mutation: *EGFR C797S* (128).

Erlotinib treatment of *EGFR* mutated and wild-type NSCLC enriches ALDH-expressing (ALDH+) stem-like cells in a NOTCH3-dependent manner, increases cell death of ALDH− cells, and increases pulmosphere-forming potential (129). Concomitant treatment with GSI and erlotinib reduces the ALDH+ subpopulation of cells in *EGFR*-mutated cell lines (129). In addition, expression of NOTCH1 and HES1 were found to be upregulated in gefitinib-resistant lung cancer, which could be reversed by NOTCH inhibition thus resulting in increased apoptosis (130). Moreover, dual targeting of EGFR and NOTCH2/3 with the CT16 antibody reduces EGFR–TKI-induced ALDH+ and RT-induced CD133+ stem cell subpopulations, the EGFR/RT-induced EMT gene signature, and expression of DNA repair genes. Combination of CT16 with RT prevents tumor regrowth of mouse xenografts. However, CT16 was not effective in treating cetuximab-/erlotinib-resistant cell lines (131). Other studies have shown that there is a differential response to GSI treatment depending on the *EGFR* status. NSCLC cell lines with undetectable EGFR protein levels are more sensitive to GSI treatment since both autophagic and apoptotic machineries are activated (132). *EGFR* T790M TKI-resistant NSCLC frequently has high IGF-1R expression levels. EGFR heterodimerizes with IGF-1R, preventing gefitinib-induced apoptosis (133). In cells overexpressing IGF-1R, combination treatment of IGF-1R inhibitors and EGFR TKIs (e.g., linsitinib and gefitinib, respectively) inhibits proliferation, increases apoptosis, and attenuates VEGF production in NSCLC cells (134). In addition, VEGF expression was found to be upregulated in *EGFR*-mutated lung adenocarcinomas which additionally increases cell survival *via* activation of AKT and STAT5 pathways (135). NOTCH1 is known to upregulate IGF-1R and its inhibition sensitizes cells to GSI-induced cell death under hypoxic conditions (107). VEGF upregulation could potentially be targeted through NOTCH-blockade-induced IGF-1R inhibition.

NOTCH activation stimulates endothelial-to-mesenchymal transition (EndMT) by downregulating endothelial markers (e.g., vascular endothelial cadherin, platelet endothelial cell adhesion molecule-1, endothelial NO synthase, and Tie1/2), upregulating mesenchymal markers [e.g., α-smooth muscle actin, fibronectin, and platelet-derived growth factor (PDGF) receptors] and migration toward PDGF-BB (136, 137). EndMT increases the production of cancer-associated fibroblasts (CAF), known to contribute to tumor progression and treatment resistance (138, 139). Podoplanin-expressing CAF have shown to be implicated in the primary resistance in NSCLC to EGFR TKI (140).

### ALK Inhibitors

Anaplastic lymphoma kinase (ALK) is a tyrosine kinase receptor which belongs to the insulin receptor family. The ALK receptor can undergo various rearrangements which have been estimated to occur in 3–7% of NSCLC patients. Currently, there are three targeted agents approved for clinical use: crizotinib, ceritinib, and alectinib. Phase I trials with crizotinib led to promising results; however, most patients develop resistance to crizotinib within 12 months due to *de novo ALK* mutations (e.g., C1156Y, L1196M, G1269A, and L1152R), *ALK* gene amplification or alternative mechanisms such as EMT or upregulation of P-glycoprotein (17). Although the next generation ALK inhibitor alectinib (CH5424802) has shown efficacy in NSCLC (141), hypoxia was found to induce resistance to ALK inhibitors crizotinib and alectinib in lung adenocarcinoma by inducing an EMT phenotype (142). Resistance to ALK inhibitors in NSCLC is mediated by mechanisms previously described to be associated with NOTCH signaling.

### Angiogenesis Inhibitors

Anti-angiogenic therapy aims at normalizing vasculature in tumors to improve blood flow and drug delivery. This can be done either by targeting VEGF (e.g., bevacizumab or ramucirumab), by targeting the DLL4 NOTCH ligand (e.g., enoticumab, demcizumab, or MEDI0639) or simultaneously (e.g., HD105). Monotherapy regimens with anti-VEGF inhibitors results in approximately 70% reduction in vasculature density and arrests blood flow (143). The surviving fraction of endothelial cells are characterized by reduced VEGFR2 and 3 reversible expression, and interstitial fluid pressure due to vasculature normalization, enabling better drug delivery to the tumor (144). Moreover, these cells show intrinsic and/or acquired resistance due to upregulation of alternative proangiogenic signals (e.g., FGF, PDGF, and TNF-α) and increase in local hypoxia (145, 146). DLL4 NOTCH ligand is partially dependent on VEGF signaling for its expression in lung tumor vessels (147). Blockade of DLL4 in glioma and breast tumors delays tumor growth even in those tumors that are resistant to anti-VEGF therapy (147). Dual targeting of DLL4 and VEGF, using the bispecific antibody HD105, inhibits tumor progression of lung adenocarcinomas and gastric cancers (148). Studies with mouse tumor cells however, have shown that overexpression of endothelial specific-DLL4/NOTCH signaling in Lewis lung carcinoma xenografts reduces primary tumor growth by reducing VEGF-induced endothelial proliferation, tumor vessel density and overall tumor blood supply. On the other hand, tumor vascular maturation and functionality was improved, and thus, drug delivery was enhanced and metastasis suppressed (149).

### KRAS-Driven Tumors

*KRAS* mutation is the most frequent oncogenic mutation (25%) in non-squamous NSCLC but no targeted therapies are available for clinical use. Several strategies are being tested in clinical trials including MEK inhibitors, focal adhesion kinase inhibitors, cyclin-dependent kinase inhibitors, and heat shock protein 90 inhibitors (150). In multicentric trials, selumetinib, an inhibitor of MEK1/MEK2 downstream of KRAS, showed no improved PFS despite extensive preclinical evidence (151). A retrospective study suggested that patients with KRAS^G12C^ tumors, prone to activate the RAS-like pathway, display shorter PFS in response to pemetrexed, while patients with KRAS^G12D^ tumors, prone to activate the PI3K pathway, show short PFS in response to gemcitabine (152). Given that NOTCH signaling is involved in the activation of both the RAS and PI3K signaling pathways, therapies targeting NOTCH in *KRAS*-driven tumors could be a promising strategy. In *in vitro* and in *in vivo* preclinical NSCLC models, GSI can increase paclitaxel sensitivity, particularly in *KRAS*-wild-type NSCLC, suggesting that *KRAS*/*BRAF* mutation status may predict combined efficacy of GSI with paclitaxel (100). Moreover, other studies have shown that GSI can suppress *KRAS*-driven NSCLC partly by suppressing ERK/MEK signaling by activating ERK phosphatase DUSP (153).

## NOTCH Targeting in NSCLC

There are several approaches to block NOTCH signaling which include GSIs, mAbs, blocking peptides, and natural compounds. GSIs prevent NOTCH receptor activation by blocking the rate-limiting step in NOTCH activation: the intramembranous cleavage by presenilin containing the γ-secretase enzyme. MAbs against the negative regulatory region of NOTCH, the ligand-binding EGF repeats in NOTCH extracellular domain, or the NOTCH ligand DLL4 in endothelial cells, block NOTCH signaling at different steps in the signaling cascade. Synthetic, stabilized, cell-permeable blocking peptides have been designed mainly to interfere with the formation of NOTCH–CSL–MAML activation complex. Natural, non-toxic compounds have gained interest since they have been shown to be associated with decreased cancer risk in lung cancer (154). Moreover, certain non-toxic agents including delta-tocotrienol (in blueberries), curcumin (in *curcuma longa* used as flavoring agent), and anthocyanidins (in berries) have shown anti-NOTCH signaling effects in NSCLC.

### Preclinical Effects of NOTCH Inhibitors

In preclinical studies, NOTCH signaling has been blocked pharmacologically by (1) GSIs: DAPT, MRK-003, PF-3084014, RO4929079, BMS-708163, LY-685458, LY-411575, and GSI XX (Table 1), (2) mAbs: CT16 (anti-EGFR and anti-NOTCH 2/3), tarextumab (anti-NOTCH 2/3), HD105 (anti-DLL4 and anti-VEGF), demcizumab (anti-DLL4), and (3) naturally occuring NOTCH signaling inhibitors: nobiletin, delta-tocotrienol, curcumin, and delphinidin (Table 2). Monotherapy usually does not render significant responses in terms of reduced proliferation, induction of apoptosis, or tumor growth delay, but NOTCH inhibition does enhance the effect of diverse chemotherapeutic agents.

**Table 1:** Outcome for preclinical trials in non-small cell lung cancer with GSIs as monotherapy or in combination with other chemotherapeutics or targeted agents.

Treatment		Outcome	Reference
GSI	Combination		
DAPT	SA	✓ Least effective clinical GSI in cleaving NOTCH receptors ✓ *NOTCH1* gain-of function/loss of function *NUMB* mutations: sensitive ✓ ↑ G1/G0 and G2/M arrest ✓ ↓ ALDH+ cells with ↑ NOTCH 1/2/3, HEY1/2 and *HESl* ✓ EGFR low/wt cells: ↓ proliferation GO/G1 arrest, ↑ Beclin-1 ✓ Reverts NOTCH-induced EMT phenotype	(74, 98, 132, 155, 156)
	Cisplatin Paclitaxel Doxorubicin Docetaxel Gefitinib Pterostilbene	✓ ↑ P-c-Jun, ↑ AP-1-regulated miR-451, ↓ MDR-1 ✓ Cisplatin-treated cells with ↑ drug transporters: sensitive ✓ ↓ Viability of cisplatin-resistant CD133+ cells ✓ Gefitinib-resistant cells: ↑ NOTCH1, HES1, and cyclin D1, ↓ P21 WAF1/CIP1 ✓ GSI + cisplatin/docetaxel/gefitinib: *in vivo* chemosensitization, ↑ G2/M arrest, ↓ proliferation, ↑ apoptosis ✓ GSI + Pterostilbene: ↓ tumor growth *in vivo*, ↓ pterostilbene-mediated ↑NICD, HES1 and ↓ PI3K/AKT, cyclin D1, survivin, DNA-PK, P-mTOR, P-S6 ribosomal protein	(87, 98, 130, 157, 158, 159)
MRK-003	SA	✓ *NOTCH1* gain-of function/loss of *NUMB* mutations: sensitive ✓ ↓ Tumor formation in H1299 stem-like cells expressing ↑ NOTCH 2/4, HES1, HEY1 resistant to cisplatin/docetaxel, rescued by N1/2 ICD (not N3 ICD) in sphere formation ✓ ↓ *NOTCH1*-mediated ↑ IGFR-1-mediated AKT-1 expression by ↓ PTEN under hypoxia and ↑ apoptosis under hypoxia ✓ ↓ NOTCH3: ↓ growth and ↑ tumor apoptosis *via* ↓ p-ERK, p-BCL-2, BCL-Xl and ↑ BIM, BAX, p-BAD proteins	(53, 74, 107, 160)
	Docetaxel Dominant neg. IGFR-1 Erlotinib	✓ GSI + docetaxel: ↓↓ tumor growth ✓ IGF-1R sensitizes cells to GSI-induced apoptosis ✓ GSI + erlotinib: ↑ ERK-regulated ↑ BIM and ↓ tumor growth	(53, 74, 107, 160)
PF-3084014	SA	✓ Preferentially ↓ NOTCH2, but also other NOTCH receptors, SPPL2b, APPC100, and APP	(155)
	Erlotinib	✓ ↓ ALDH+ NOTCH3-dependent cells in *EGFR*-mutated cell lines ✓ EGFR negatively regulates Notch activity *via* its TK activity	(129)
RO4929079	SA	✓ Preferentially ↓ NOTCH1 followed by NOTCH2/3, SPPL2b and APPC100	(155)
	Erlotinib	✓ ↓ miR-223, CD44+ erlotinib-resistant cells ✓ ↑ FBXW7 and reverses erlotinib-resistance	(161)
BMS-708163	SA	✓ ↓ NOTCH1, HES1, PI3K, and AKT (but not mTOR) and Ki67 ✓ ↑ G1 arrest, active caspase 3 and PARP	(162)
	Gefitinib	✓ ↓ 3D colony growth, Ki67, gefitinib-resistant tumor xenograft growth ✓ ↑ Cytotoxicity and apoptosis	
LY-685458	SA	✓ ↓ NOTCH, DLK1-induced ↑ MMP9 expression, invasion	(163)
LY-411575	DDR1 inhibitor 7rh	✓ Additive tumor growth delay of *KRAS*-driven (including *TP53*-null) PDX NSCLC and ↑ apoptosis ✓ Similar therapeutic efficacy to cisplatin/paclitaxel, but displayed coagulative necrosis, ↓ p-AKT and p-p38	(164)
GSI XX	YC-1 HIF inh RT	✓ RT-induced HIF-1α ↑ NOTCH3 under hypoxia (reversed by YC-1) ✓ GSI XX 24 h post YC-1 + 8 Gy: strongest tumor growth delay *in vivo*	(165)
GSI?	Cisplatin ABT-737 (BH3-only mimetic)	✓ ↓ NOTCH3: ↓ the cisplatin-mediated ↑ in spheroid forming efficiencies, LC3 and ↓ ALDHA1, CD44 ✓ GSI + ABT-737: synergistic ↓ proliferation, tumor growth *in vivo* and ↑ BIM and cleaved PARP	(88, 166)

*SA, single agent; ↑, increase/upregulated; ↓, decrease/downregulated; GSI, gamma-secretase inhibitor; GSI?, unspecified GSI; RT, radiotherapy; neg., negative; EGFR, epidermal growth factor receptor; IGF-1R, insulin-like growth factor 1 receptor; DDR1, discoidin domain receptor 1; SPPL2b, signal peptide peptidase-like 2b; APPC100, C-terminal 100 amino acids of amyloid precursor protein; APP, amyloid precursor protein; PI3K, Phosphoinositide 3-kinase; PARP, poly ADP-ribose polymerase 1; DLK1, delta-like non-canonical NOTCH ligand 1; MMP9, matrix metallopeptidase 9*.

**Table 2:** Outcome for preclinical trials in non-small cell lung cancer with mAbs or natural NOTCH inhibitors (inh) alone or in combination with other chemotherapeutics or targeted agents.

Treatment			Outcome	Reference
NOTCH-based		Combination		
mAb	CT16 (anti-EGFR and anti-NOTCH 2/3)	SA	✓ ↓ Tumor-initiating capacity upon reimplantation, tumor growth and reversal of EMT phenotype ✓ Cetuximab and erlotinib-resistant cell lines: not effective	(131)
		RT	✓ ↓ RT-enriched CD133+, EGFR inh-enriched ALDH+ ✓ ↓ The RT-induced EMT (upregulated in CD133+ but not in ALDH+ cells) and DNA repair genes ✓ Prevented tumor regrowth, delayed acquired resistance to EGFR inhibitors	(131)
	Tarextumab (anti-NOTCH 2/3)	SA	✓ ↓ Tumor-initiating capacity upon reimplantation	(131)
		Erlotinib Cetuximab	✓ Additive effect on NOTCH3+ NSCLC PDX	
	HD105 (anti-DLL4 and anti-VEGF)	SA	✓ ↓ Cell proliferation, vessel sprouting and ↑ apoptosis ✓ ↓ Tumor progression *in vivo*	(148)
	Murine anti-DLL4	SA	✓ ↑ CD31+ tumor vessel density ✓ ↑ Thin and more branched vessels	(148)
		Murine anti-VEGF antibody	✓ ↓ Tumor vessel density and functionality	
	Demcizumab (anti-DLL4)	Abl, Src, c-Kit, and DDR1 inh. Dasatinib	✓ Durable and better therapeutic efficacy than cisplatin/paclitaxel in orthotopic PDX *KRAS*-driven lung adenocarcinomas	(164)
Natural inh	Nobiletin (citrus peels)	SA	✓ ↑ miR-200b under hypoxia ✓ ↓ NOTCH1, JAGGED1/2, HES1 and HEY1 (but not NOTCH2/3, nicastrin: presenilin 1/2 and APHl) independent of gamma-secretase, ↓ invasion in Matrigel ✓ Reversal of hypoxic-induced EMT phenotype	(167)
	Delta-tocotrienol (blueberries)	SA	✓ ↓ NOTCH1, colony formation and invasiveness ✓ ↑ miR-34a G0–G1 arrest, apoptosis *via* TP53 activation	(168)
		Cisplatin	✓ Potentiates antitumorigenic effect ✓ ↑ NF-κB DNA binding activity ✓ ↓ NOTCH1, HES1, Bcl-2, cleaved caspase-3 and PARP	(168)
	Curcumin (ginger and other plants)	SA	✓ ↓ NOTCHl by ↓ EZH2 *via* miR-let7c and miR-101 ✓ Delays tumor growth and prevents metastasis	(121)
	Delphinidin (berries)	Bilberry anthocyanidins	✓ ↓ Cell proliferation and migration: ↓ NF-κB, NOTCH1, β-catenin, c-MYC, MMP9, Cyclin D1 and B1 ✓ ↑ G2/M arrest, apoptosis ✓ ↓ Tumor growth *in vivo*	(169)

*SA, single agent; ↑, increase/upregulated; ↓: decrease/downregulated; NSCLC, non-small cell lung cancer; RT, radiotherapy; EGFR, epidermal growth factor receptor; DDR1, discoidin domain receptor 1; PDX, patient-derived xenograft; VEGF, vascular endothelial growth factor; EMT, epithelial–mesenchymal transition; PARP, poly ADP-ribose polymerase; EZH2, enhancer of zeste homolog 2; APH1, anterior pharynx-defective 1; MMP9, matrix metalloprotease 9; mAbs, monoclonal antibodies*.

Despite being one of the least potent GSIs in preclinical studies (155), DAPT has proven to be efficacious as single agent in treating NSCLC with altered NOTCH signaling pathway members (74), in inducing apoptosis and autophagy, preferentially in cells expressing EGFR wild type or without EGFR expression (132), by inducing cell cycle arrest in G1 and G2/M phases (98), and reducing the ALDH+ stem cell population (156). In addition, DAPT can enhance the effects of cisplatin by reducing the CD133+ stem cell subpopulation (157). DAPT also prevents cross-resistance to paclitaxel and doxorubicin (87), of docetaxel by decreasing the AP1/miR-451-induced MDR-1 expression (98), of gefitinib by reverting the EMT phenotype (130, 158), and of pterostilbene by preventing PI3K/AKT activation (159). Several other GSIs have shown to be able to attenuate EGFR–TKI resistance to erlotinib: MRK-003 *via* increase of the ERK-regulated pro-apoptotic BIM (160), PF-3084014 through downregulation of ALDH+ stem cells (129), and RO4929079 by decreasing CD44+ stem cells and increasing the miR-223-induced decrease in FBXW7 expression (161). In addition, BMS-708163 can revert resistance to gefitinib in NSCLC (162). MRK-003 has shown therapeutic enhancement of docetaxel (53) and IGF-1R inhibition by inducing apoptosis (107). GSI XX enhances therapeutic efficacy of the combination including a HIF-targeting small molecule inhibitor (YC-1) and radiotherapy, but only when added after the combination (165). GSI XX also improves the response of cisplatin by promoting autophagy, reducing the ALDHA1+ and CD44+ stem cells (88), or when combined with a BH3-mimetic (ABT-737) by inducing apoptosis in a Bim-dependent manner (166). The doses used in these studies have been summarized in Table 3.

**Table 3:** Comparison between preclinical *in vivo* doses of NOTCH-targeted agents used for non-small cell lung cancer treatment.

Treatment		Preclinical dose	Reference
Type	Name		
GSI	DAPT	8 mg/kg ip 3 days/week or l0 mg/kg ip once every 3 days * 6 injections	(130, 159)
	MRK-003	150 mg/kg 3 days/week	(160)
	BMS-708163	10 mg/kg po 5 days/week	(162)
	LY-411575	3 mg/kg po daily	(164)
	GSI XX	200 µg/kg ip 3 days/week * 2 cycles	(165)
	GSI?	200 µg/kg ip 3 days/week * 2 cycles	(166)
mAb	CT16 (anti-EGFR and anti-NOTCH 2/3)	40 mg/kg	(131)
	Tarextumab (anti-NOTCH 2/3)	40 mg/kg	(131)
	HD105 (anti-DLL 4 and anti-VEGF)	3.25 mg/kg ip 1–2 days/week or 6.5 mg/kg ip 1 day/week	(148)
	Murine anti-DLL4	2.5 mg/kg ip 1–2 days/week	(148)
	Demcizumab (anti-DLL4)	10 mg/kg ip 1 day/week	(164)

*GSI, gamma-secretase inhibitor; mAb, monoclonal antibody; ip, intraperitoneal; po, *per os* (oral administration)*.

Monoclonal antibodies against DLL4 used to target the tumor vasculature, result in an increase of branched vessels but with decreased functionality. Combination therapy with bevacizumab increases apoptosis and decreases vessel branching and tumor progression (148). One of the major problems with anti-VEGF therapy in the clinic is the increased levels of hypoxia leading to more aggressive treatment-resistant tumor cell populations, but it also leads to hemoptysis, hypertension, and arterial thrombus embolism due to effects on the normal vasculature in the heart, endocrine and nervous systems (170, 171). Because these anti-angiogenic inhibitors are not tumor specific, successful clinical implementation will most likely consist of the use of drug doses/scheduling aiming at vessel normalization, promoting formation of functional vessels to improve drug delivery (172), or in their combination with other therapies, rather than those aiming at tumor regression. Tarextumab in combination with erlotinib, cetuximab, or CT16, increases therapeutic efficiency of EGFR targeting and delays acquired resistance by similar mechanisms as those with TKI therapy (131).

Natural-occurring compounds have shown remarkable anti-NOTCH effects not only by affecting the expression of NOTCH pathway members (NOTCH1 and HES1) which results in reversal of the hypoxic-induced EMT phenotype (167), but also by upregulation of miR-34a, TP53 and apoptosis (168); inhibition of EZH2 (121, 122); and have shown to be able to potentiate antitumorigenic effects in combination with cisplatin (168).

**Figure 1:**
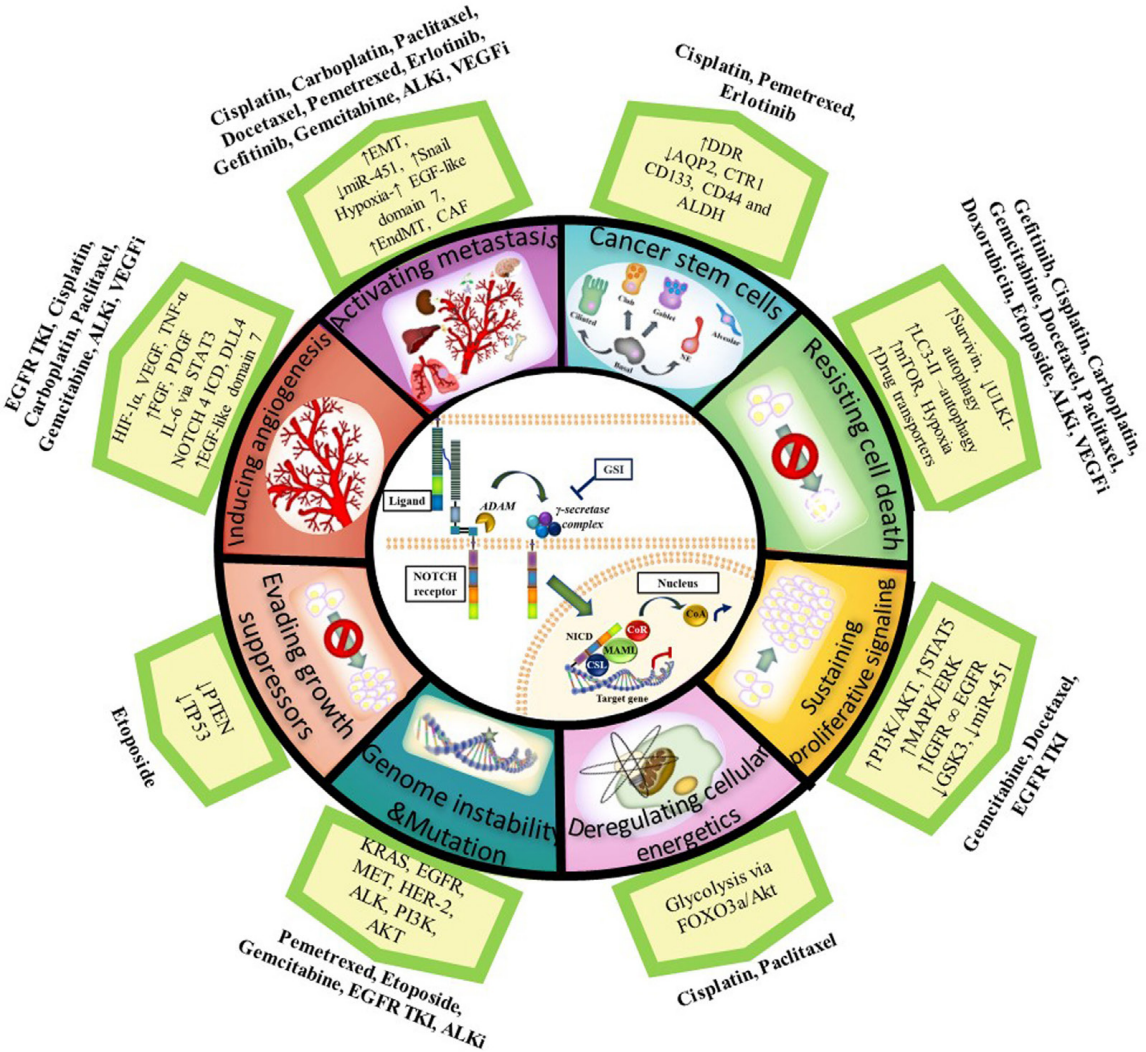
Notch and the Hallmarks of Cancer in tumor resistance to chemotherapy and targeted agents in NSCLC. NOTCH1 *sustains proliferative signaling* by upregulating PI3K/AKT pathway *via* PTEN repression and induction of IGF-1R under hypoxia. PI3K/AKT and MAPK/ERK/RAS then upregulate HIF-1α in an oxygen-dependent manner. HIF-1α binds to N1 ICD to regulate each other: HIF-1α increases gamma-secretase activity to activate NOTCH signaling whereas factor inhibitor HIF (FIH) hydroxylates and downregulates NICD activity. NOTCH1 upregulates IGF-1R which forms a heterodimer (∞) with EGFR and increases survivin (apoptosis inhibitor) to *resist cell death*. Cancer cells also resist cell death by upregulating the autophagosomal marker LC3 and/or drug transporters (ABCB1 and ABCG2) mediated by NOTCH-dependent AP1/microRNA-451 or through a glycolysis-associated mechanism *via* FOXO3a/AKT signaling thus promoting *deregulation of cellular energetics*. CSL binds to TP53 and they can both repress each other thus *evading growth suppressors*. NOTCH signaling also has a role in the maintenance of cancer stem cells (CSCs). Chemotherapy induces an enrichment of resistant tumor cells expressing CSC markers (CD133, ALDH, CD44). CSCs have downregulated the AQP2 and CTR1 drug transporters which prevent drug accumulation and reduce dsDNA damage. In addition, CSCs have increased DDR and repair pathways. NOTCH facilitates *metastasis* by increasing the epithelial–mesenchymal transition (EMT) *via* an increase in TWIST, SNAIL, SLUG, and ZEB. NOTCH activation can also stimulate endothelial-to-mesenchymal transition (EndMT) which increases the production of cancer-associated fibroblasts (CAF) which are known to be involved in chemotherapy resistance. DLL4 ligand is positively regulated by proangiogenic factors (e.g., VEGF-A, bFGF), IL-6 mediated by STAT3 activation, FOXC protein, N4 ICD, and HIF-1α to *induce angiogenesis*. DLL4 downregulates VEGFR2 to inhibit VEGF-A and endothelial cell proliferation and migration. DLL4 ligand targeting inhibits tumor progression of human lung adenocarcinomas. Upregulated NOTCH signaling activity has been found in cancers with other genetic alterations/mutations [*KRAS, EGFR, HER2, MET*, anaplastic lymphoma kinase (*ALK*), *PI3K*] with which it cross-talks. Abbreviations: I, inhibitor; DDR, DNA-damage response; miR, microRNA; GSI: gamma-secretase inhibitor; dsDNA, double stranded deoxyribonucleic acid; N4 ICD, NOTCH 4 intracellular domain.

As described in this review, the NOTCH signaling pathway acts upon different aspects of the hallmarks of cancer described by Hanahan and Weinberg to promote cancer resistance, and its targeting can enhance the effect of chemotherapeutics or agents targeting oncogenic driver mutations when used in combination (see Figure 1 for summary). Importantly, several studies have shown that NOTCH inhibition in combination with radiation therapy also improves outcome in NSCLC (165, 173–175). Since most patients receive combinations of radiotherapy and chemotherapy, these results are significant and show there is great potential for combining NOTCH inhibitors with radiotherapy and chemotherapy to target the CSCs and reduce treatment resistance. Further analysis on the implications of combining NOTCH-based targeted therapy with radiotherapy have been recently described (76, 174, 175).

### Clinical Trials Using NOTCH Pathway Inhibitors

Only a few of the preclinically tested NOTCH inhibitors have progressed into clinical trials in NSCLC patients including BMS-906024, PF-3084014, RO4929079, MK-0752, LY900009, LY3039478, BMS-986115, enoticumab, demcizumab, and MEDI0639 (Tables 4 and 5). Clinical studies using NOTCH inhibitors as monotherapy have shown limited effect on local control and some have been halted (176, 177). Nevertheless, conclusions on GSI effects in cancer patients are debatable since patients are not pre-screened for NOTCH signaling upregulation/mutation and different clinical GSIs have diverse potencies, specificities, and side effects (155).

**Table 4:** Maximum tolerated dose (MTD) and recommended phase 2 doses (RP2D) for NOTCH-based therapies in clinical trials for advanced or metastatic solid tumors who no longer respond to or have relapsed from standard therapies.

Treatment		MTD	RP2D	Type of cancer tested	Clinical trial ID
Type	Name				
GSI	PF-03084014	220 mg po b.i.d.	150 mg po b.i.d.	Lung, colon, desmoid, breast, thyroid, endometrial, leiomyosarcoma, pancreas, and liver	(178)
	RO4929097	NR (MDT: 20 mg 3 days/week)	20 mg 3 days/week	NSCLC, sarcoma, neuroendocrine, SCC, head and neck, pancreas, breast, colorectal high-grade glioma, renal, ovarian, gastrointestinal, stromal, melanoma, hepatocellular, endometrial, and cholangiocarcinoma	(179); PJC-004/NCI 8503 (180)
	LY900009	30 mg 3×/week	UD (<30 mg 3×/week)	NSCLC, colorectal, endometrial, ovarian, pancreatic, sarcoma, papillary adenocarcinoma, and leiomyosarcoma	NCT01158404 (181)
	LY3039478	ONG (MDT: po Q12H)		Advanced solid tumors	NCT02836600; NCT02784795^a^
	BMS-906024	ONG (MDT: 6 mg iv QW)		NSCLC, triple-negative breast cancer, and tumors with proven active NOTCH	NCT01292655, NCT01653470
	MK-0752	UD (MDT: 4,200 mg po daily)	(UD dose >1,800 mg) po QW	NSCLC, high-grade glioma, glioblastoma multiforme, anaplastic astrocytoma, meningioma, mesothelioma, oligoastrocytoma, oligodendroglioma, leiomyosarcoma, bladder, breast, colorectal, kidney, endometnal, gastrointestinal, head and neck, melanoma, ovarian, pancreas, sarcoma, thyroid, urothelial, and stromal	(182, 183)
	BMS-986115	UD (MDT: QD)		Advanced solid tumors	NCT01986218
mAb anti-DLL4	Enoticumab	NR	4 mg/kg Q3W or 3 mg/kg Q2W	Lung, NSCLC bronchoalveolar-type, colorectal, ovarian, pancreatic, sarcoma, breast, salivary gland, head and neck, thyroid, prostate, cholangiocarcinoma, and hepatocellular	(184)
	Demcizumab	NR	5 mg/kg Q2W (with cardio-protective agents)	NSCLC, SCLC, colorectal, renal, pancreatic, salivary gland, breast, sarcoma, kidney, melanoma, head and neck, gastric, prostate, bladder, esophageal, ovarian, testicular, penile, and mesothelioma	(185)
	MEDI0639	NR	Lack of clinical activity	Advanced solid tumors	NCT01577745

*Life expectancy ≥ 3 months*.

*SCC, squamous cell carcinoma,; NSCLC, non-small cell lung cancer; SCLC, small cell lung cancer; po, *per os* (oral administration); iv, intravenous; b.i.d., bis in die (=2×/day); NR, not reached; MDT, max dose tested; UD, undocumented; ONG, ongoing; Q12H, every 12 h; QW, once every week; QD, quaque die (once per day); Q3W, once every 3 weeks; Q2W, once every 2 weeks*.

*^a^Selected patients with gene/protein alterations in NOTCH pathway*.

**Table 5:** Outcome and on-target effects of NOTCH-targeted therapies in clinical trials where lung cancer patients have been included.

Treatment			Outcome	On-target effects	Clinical trial ID
NOTCH-based		Combination			
GSI	PF-03084014	SA	ORR 13%: 46 patients 1 CR (adv. thyroid cancer) 5/7 PR (desmoid tumor) Duration: 1.74–24 months	↓ HES4 in peripheral blood at RP2D	(178)
	RO4929097	SA	5/5 DP	IC	NCT01193868
		VEGFR inh cediranib or mTOR inh Temsirolimus	1 PR at best 11/17–19 SD 7/19 DP PFS: 4.2 months	No ∞ NOTCH biomarkers and time to progression at RP2D	(179); PJC-004/NCI 8503 (180)
	LY900009	SA	No OR 5/35 SD (NSCLC; papillary adenocarcinoma, leiomyosarcoma, ureter, and rectal carcinoma)	↓ Aβ in plasma 1/35 ↑ glandular mucin	NCT01158404 (181)
	MK-0752	SA	1 CR ≥ 1 year (anaplastic astrocytoma) 12 SD ≥ 4 months (high-grade glioma) No clinical activity for extracranial tumors	↓ NOTCH in hair follicles (l,800–4,200 mg po 1×/week)	(182)
		Dalotuzumab anti-IGFR1	12/12 DP	UD	(183)
mAb anti-DLL4	Enoticumab	SA	2/44 PR (NSCLC bronchoalveolar-type and ovarian cancer) 16/44 SD (3 patients ≥ 6 months) 1 DP (low titer anti-enoticumab antibodies)	In 39/40 tumors ▪ DLL4+ and CD31+ ▪ 30% VEGFR2+ vessels ∞ N1 and N3 ICD in tumor, NOTCH3 and DLL4 in vessels ▪ No ∞ NOTCH biomarkers and time to progression	(184)
	Demcizumab	SA	21/48 SD (NSCLC, renal and colorectal carcinomas) 16/25 (10 mg/kg): SD or PR 6/55 anti-demcizumab antibodies. No impact on biological activity	In blood: ▪ ↓ NOTCH and WNT pathway genes (*HEY1/2, SPON2, CCNA2*) ▪ ↑ Negative regulators (*AP2A1, AP2B1, SFRP2*: *FOXO3, CDH1ANK1, BCL2L1*) angiogenesis genes In hair follicles: ▪ ↓ Stem cell genes ▪ ↑ vascular genes	(185)
	MEDI0639	SA	1 PR for 1.3 months 9/25 SD for 5.9 months 12/25 DP PFS ≤ 7.1 months OS ≤ 23.9 months	IC	NCT01577745

*GSI, gamma-secretase inhibitor; mAb, monoclonal antibody; inh, inhibitor; SA, single agent; ↑, increase/upregulated; ↓, decrease/downregulated; ORR, objective response rate; OR, objective response; CR, complete response; PR, partial response; DP, disease progression; adv., advanced; RP2D, recommended phase 2 dose; IC, inconclusive; SD, stable disease; PFS, progression-free survival; OS, overall survival; ∞, association; Aβ, amyloid-β peptide; NSCLC, non-small cell lung cancer; po, *per os* (oral administration); UD, undocumented; N1/3 ICD, NOTCH 1/3 intracellular domain; SPON2, spondin 2 extracellular matrix protein; CCNA2, Cyclin A2; AP2A1, adaptor related protein complex 2 subunit alpha 1; AP2B1, adaptor related protein complex 2 subunit beta 1; SFRP2, secreted frizzled related protein 2; FOXO3, forkhead box protein O3; CDH1, cadherin 1; ANK1, ankyrin 1; BCL2L1, B-cell CLL/Lymphoma 2 like 1*.

In the clinical trials including lung cancer patients, the best responses using GSI were obtained with LY900009 as single agent (NCT01158404) with which 5/35 stable diseases were accomplished. GSI efficacy was confirmed by measuring on-target undesirable effects of NOTCH inhibition. Low clinical activity was explained by fast drug absorption and elimination (181). Combination of RO4929097 with cediranib or temsirolimus (PJC-004/NCI8503) only marginally improved outcome, obtaining at best one partial response and 11/17–19 stable diseases with a median PFS of 4.2 months (179, 180). In the latter studies, there was no association between NOTCH biomarkers and time to progression at the recommended phase II dose.

Monoclonal antibodies against DLL4 have shown better outcomes compared with GSI treatment. DLL4 has been shown to be both important for tumor vasculature as well as for maintaining tumor-initiating capacity of tumor stem cells in various tumor models (186). As single agents (NCT01577745), enoticumab, demcizumab, and MEDI0639 may have up to two partial responses (enoticumab, MEDI0639) and between 36 and 44% of stable diseases lasting for an average of 6 months (184, 185). Blood of demcizumab-treated patients presented a decrease in NOTCH and WNT pathway members, and an increase of their negative regulators, whereas the hair follicles had a downregulation of stem cell genes, and an upregulation of vascular genes. In enoticumab-treated patients however, there was no association between NOTCH biomarkers and time to disease progression. Clinical trials combining demcizumab with pemetrexed and carboplatin for non-squamous NSCLC (NCT01189968) obtained objective responses in 50% of patients, demcizumab-target efficacy was observed in the blood, and a demcizumab dose was recommended for phase II trials (187). Phase II trials (YOSEMITE) of demcizumab in combination with paclitaxel and gemcitabine to treat metastatic pancreatic cancer however, were recently discontinued because primary efficacy endpoints (PFS) were not significantly better than placebo (chemotherapy alone) however, results on other trials (PINNACLE, DENALI) are yet to be evaluated. It remains to be assessed how combination therapy with demcizumab would influence survival in NSCLC patients.

### Side Effects of NOTCH Therapeutics

*Gastrointestinal toxicity* is an undesired on-target effect of γ-secretase inhibitors due to simultaneous inhibition of NOTCH1 and 2, which have redundant roles in regulating homeostasis in the crypt epithelium, resulting in excessive secretory differentiation and goblet cell metaplasia (188–192). GSIs used preclinically (Tables 3 and 6) result mainly in gastrointestinal toxicity, as seen for MRK-003 (193), PF-3084014 (194), LY-411575 (192, 195), DBZ (188, 190, 196), and compound E (197). These effects are usually found in combination with severe body weight loss but can be mitigated using glucocorticoids (dexamethasone) as shown for PF-3084014 (194) and DBZ (198). The use of monoclonal antibodies, such as OMP59R5, minimizes the associated intestinal toxicity (124) probably because NOTCH1, one of the major contributors of normal gastrointestinal architecture, is not targeted. No gastrointestinal effects were detected for anti-DLL4 antibodies (199), which is explained by redundancy with DLL1 (200), nor for natural agents, except for curcumin where loose bowel movements were reported (201). In clinical trials (Tables 4 and 7), several studies reported severe gastrointestinal toxicities (grade III or higher) including diarrhea, nausea, dehydration and mucositis for RO4929097 (179, 180), LY900009 (181), and MK-0752 (183) (Table 7). However, these effects were mitigated with adequate intermittent scheduling (182). Oral dosing of PF-03084014 twice a day resulted in manageable gastrointestinal toxicity, better than that for RO4929097 and MK-0752 (178), indicative that not all GSIs are equally potent nor biological equivalents because they target different NOTCH receptors and also have diverse off-target effects (155). In clinical trials (NCT01577745), only abdominal pain was reported for enoticumab (184), and gastrointestinal toxicity was milder than for GSI treatment in general (185).

**Table 6:** Associated toxicities of NOTCH-based therapies used preclinically for non-small cell lung cancer.

Treatment		Dose	Toxicity	Reference
Type	Name			
GSI	MRK-003	100 mg/kg po 3 days	✓ Diarrhea, dehydration, severe weight loss	(193)
	PF-3084014	150 mg/kg po 7 days	✓ Goblet cell hyperplasia ✓ ↓ Total whole blood count, lymphocytes, basophils, eosinophils, leukocytes ✓ Mild jejunal eosinophilic inflammation	(194)
		100 mg/kg GSI, 3 weeks * 2 cycles; 1.0 mg/kg dexamethasone on weeks 1 and 3	✓ Dexamethasone ameliorates gastrointestinal effects (DLT)	
	LY-411575	1–10 mg/kg po 5 or 15 days	✓ Altered lymphocyte development, thymus atrophy, ↓ body weight ✓ ↑ Goblet cell number, secretion of mucin into the intestinal lumen, epithelial erosion, infiltration of inflammatory cells in the lamina propria and necrosis ✓ Skin: epidermal/epithelial hyperplasia, follicular/epidermal inclusion cysts	(192, 195)
	GSI	10 µmol/kg 1 day	✓ Gastrointestinal mucous metaplasia, conversion of cryptal cells into goblet cells (goblet cell hyperplasia)	(197)
	DBZ	✓ 2.5 mL/kg ip 5 days ✓ 3–30 µmol/kg ip 5 days ✓ 10 µmol/kg daily 4 weeks	✓ Distension of the stomach and intestine ✓ ↑ Mucous, goblet cell metaplasia (duodenum, jejunum) and hyperplasia, apoptosis (small intestinal crypt epithelial cells, large intestinal glands), villus atrophy, and severe diarrhea ✓ Splenic marginal zone lymphoid tissue atrophy	(188, 190, 196)
		DBZ 10 µmol/kg ip Dexamethasone 15 mg/kg ip Daily 5 or 10 days	✓ DBZ: ↑ intestinal secretory metaplasia, goblet cell hyperplasia and *↓* proliferation small intestine crypt cells ✓ Combination: normal goblet cell numbers and tissue architecture of intestinal epithelium	(198)
mAb	OMP59R5 (anti-NOTCH2/3)	40 mg/kg every other day	✓ Minimal intestinal toxicity ✓ Rodent teeth affectance (long-term repeated high doses)	(124)
	HD105 (anti-DLL4 and anti-VEGF)	1–3 mg/kg QW for 8 weeks 10 mg every 3 days (5 doses, 30 mg/kg)	✓ ↑ Activation of endothelial cells ✓ Sinusoidal dilation ✓ Centrilobular hepatocyte atrophy	(202)
	Anti-DLL4	10 mg/kg 2 weeks	✓ No impact on intestinal goblet cell differentiation	(199)
Natural agents	Delta-tocotrienol (blueberries)	200–800 mg/kg sc 14–30 days	✓ Dose-dependent severity (up to moderately severe) of dermatitis and inflammation ✓ No adverse effects were observed in any tissues or organs	(203)
	Curcumin (ginger and other plants)	iv 14 days: ✓ 250 mg/kg ✓ 500–1,000 mg/kg	✓ 250 mg/kg: piloerection and minor weight loss ✓ 500 mg/kg: ↓ spontaneous motility and bowel movements, piloerection and ↓ weight (in 3 mice) ✓ 1,000 mg/kg: lethal within 1 h of administration, respiratory distress, bradypnea, and paralytic gait	(201)

*GSI, gamma-secretase inhibitor; mAb, monoclonal antibody; ip, intraperitoneal; po, *per os* (oral administration); sc, subcutaneous; iv, intravenous; QW, once every week; ↑, increase/upregulated; ↓, decrease/downregulated; DLT, dose-limiting toxicity*.

**Table 7:** Toxicity and pharmacokinetics (PK) of NOTCH-targeted therapies in clinical trials where lung cancer patients have been included.

Treatment			Toxicity	PK	Clinical trial ID
NOTCH-based		Combination			
M	PF-03084014	SA	Manageable gastrointestinal adverse events^a^	T_1/2_: 22–40 h after-multiple dosing.	(178)
	RO4929097	SA	Serious adverse events in ≥1/5 patients: ▪ Small intestine obstruction, constipation, nausea ▪ Lung infection/sepsis, dyspnea ▪ Cardiac arrest, tachycardia	UD	NCT01 19386; NCT01217411
		VEGFR inh cediranib	Grade III–IV: diarrhea, headache, hypertension, nausea, hypothyroidism, hypophosphatemia	Combination did not affect PK profile	(179), PJC-004/NCI 8503
		mTOR inh temsirolimus	Grade III: rash, mucositis	RO4929097 induces CYP3A4: ↑ temsirolimus CL	(180)
	LY900009	SA	Grade III: mucosal inflammation	Absorption: 1–4 h Elimination *T*_1/2_: 2–4 h LSN2831047 (GSI metabolite): appearance: 2–6 h, *T*_1/2_:5–14 h	NCT01158404 (181)
		SA	Weekly dosing was generally well tolerated	Slow absorption half-life: 15 h	(182)
	MK-0752	Dalotuzumab anti-IGFR1	Grade III dehydration, rash, and diarrhea	▪ MK-0752 1.63–8 μmol/L in plasma ▪ Dalotuzumab 34–64 μg/mL in serum (at day 8), accumulated in time	(183)
mAb anti-DLL4	Enoticumab (^b^)	SA	Grade III (0.5 mg/kg Q3W): nausea Grade III (1 mg/kg Q2W): abdominal pain Severe effects in 4 patients: ventricular dysfunction and pulmonary hypertension	Nonlinear PK *T*_1/2_: 8–9 days Dose-independent CL (dose > 1.5 mg/kg) > 2 mg/1 in plasma ∞ max. tumor activity	(184)
	Demcizumab	SA	Generally well tolerated at doses ≤ 5 mg weekly. 4 patients (10 mg/kg Q2W): congestive heart failure Not more than one DLT per dose level	PK within linear range CL: 4.17 mL/day/kg *T*_1/2_:15.9 days (>10 μg/mL)	(185)
	MEDI0639	SA	No participants with DLT	AUC: 7.4–512 μg/day/mL [Conc]_Max_; in blood: 3.2–81.6 μg/mL CL: 1.4–0.5 L/day *T*_1/2_: 1.5–8.25 days	NCT01577745

*GSI, gamma-secretase inhibitor; mAb, monoclonal antibody; inh, inhibitor; SA, single agent; ID, identifier; *T*_1/2_, half-life; UD, undocumented; CL, clearance; ∞, association; Q3W, once every 3 weeks; Q2W, once every 2 weeks; DLT, dose-limiting toxicity; [Conc]_Max_, maximum observed concentration; AUC, area under the concentration–time curve; CYP3A4, cytochrome P450 3A4*.

*^a^Better symptoms than those for RO4929097 and MK-0752*.

*^b^Better gastrointestinal toxicity than GSI*.

*Skin* adverse effects occur after GSI treatment due to the physiological function of NOTCH signaling in keratinocyte differentiation (195). In preclinical studies (Table 6), NOTCH inhibition may cause epidermal and epithelial cell hyperplasia, and follicular and epidermal inclusion cysts (192, 195). Natural inhibitors of NOTCH signaling showed mild to moderate dermatitis at the site of injection for delta-tocotrienol (203) and piloerection for high doses of curcumin (201). In patients (Table 7), skin toxicity (grade III or higher) has been reported for GSI (but not for anti-DLL4 antibodies) and is attributed exclusively to skin rash for RO4929097 (179, 180) and MK-0752 (183).

In the *immune system*, NOTCH regulates megakaryocyte development and the myeloid and erythroid lineages (204). In preclinical studies (Table 6), NOTCH inhibition may lead to mild eosinophilic inflammation, leukopenia, lymphocytopenia, altered lymphocyte development, thymus atrophy, and/or splenic marginal zone lymphoid tissue atrophy for PF-3084014 (194), LY-411575 (192, 195), and DBZ (188). In patients (Table 7), no severe toxicities (grade III or higher) were reported.

In the *vasculature*, there are also toxicities reported in preclinical studies (Table 6), accounting for pathological activation of endothelial cells and sinusoidal dilation for HD105 (202). In patients (Table 7), there were toxicities (grade III or higher) reported accounting for cardiac arrest, tachycardia, ventricular dysfunction, congestive heart failure for the GSI RO4929097 (NCT0119386; NCT01217411), for enoticumab (184), and for demcizumab (185).

In the *lung*, there were generally no effects reported preclinically (Table 6) except for respiratory distress at very high doses of curcumin (201). In patients (Table 7), however, toxicities (grade III or higher) included lung infection/sepsis, dyspnea, and pulmonary hypertension for RO4929097 (NCT0119386; NCT01217411) and for enoticumab (184).

### NOTCH Inhibition in Lung Cancer Comorbidities

Lung cancer patients often present several comorbidities. When considering the potential for NOTCH/γ-secretase inhibitors, normal tissue effects are usually dose-limiting. The potential role of NOTCH inhibition has been investigated not only in lung cancer but also in other lung pathologies, such as pulmonary goblet cell metaplasia, lung fibrosis, allergic asthma, chronic inflammation, and pulmonary arterial hypertension (PAH). The pathophysiology of pulmonary goblet cell metaplasia is similar to that of cystic fibrosis, bronchitis, and asthma. NOTCH inhibition may be of potential benefit in such cases by reducing the number of secretory cells and altering basal cell differentiation in adult lung toward a ciliated phenotype (205). In lung fibrosis, characterized by fibroblast proliferation leading to excess extracellular matrix deposition (collagen and glycosaminoglycans) and tissue remodeling occurring frequently 6–9 months after radiation treatment, GSI prevents NOTCH/JAGGED1-induced myofibroblast differentiation in response to frizzled class receptor 1 (FZZ1) by decreasing the expression of alpha-smooth muscle actin (α-SMA) (136, 206). Asthma and chronic inflammation (which may occur after radiation treatment) are characterized by airway hyperresponsiveness and enhanced immune response. NOTCH receptors are expressed in the surface of mature lymphocytes whereas NOTCH ligands are present in antigen-presenting cells. Inhibition of airway hyperresponsiveness and inflammation by repressing Th2-mediated IL-4 production can be observed with the inhibition of NOTCH on CD4+ T-cells and JAGGED1 on bone marrow dendritic cells (207). Pulmonary veno-oclusive disease (PVOD), an uncommon cause of PAH, may appear after combined therapy consisting of surgery and mitomycin with perioperative chemotherapy. PVOD requires upregulation of NOTCH3, smooth muscle cell proliferation in small pulmonary arteries, and increased vascular resistance to develop. PAH severity correlates with NOTCH3 and its downstream effector HES5. Thus, targeting the NOTCH3–HES5 axis with GSIs may improve PAH treatment (163).

## Conclusion and Perspectives

Non-squamous NSCLC is the most common form of lung cancer and a deadly disease. Despite detailed knowledge on tumor driver mutations and multimodal treatment regimens with surgery, and systemic treatment using chemotherapeutics, targeted agents, immune therapy, and radiotherapy, tumor resistance, relapse and dose-limiting toxicities are common. NSCLC is a highly heterogeneous disease at different levels and is constantly striving for survival by acquiring new favorable pro-survival mutations. There is mounting evidence implicating CSCs, and the dynamically changing tumor microenvironment, as the drivers of tumor heterogeneity which in turn results in tumor progression, metastasis, and therapeutic resistance. CSCs provide an interesting therapeutic targeting opportunity to tackle tumor resistance. There is paramount evidence for a role of the NOTCH signaling pathway in driving tumor resistance through cancer (stem) cells and cross-talk with other pathways. We identified roles for NOTCH signaling in mechanisms of tumor resistance mediated *via*, mainly, ABC drug transporters, EMT, hypoxia, pro-survival pathways, and VEGF, among others (Figure 1).

Monotherapy treatment using NOTCH inhibitors, similarly to what happens for other therapies, is not sufficient to induce tumor control or cure. Because not all patients have an aberrantly high NOTCH expression, and NOTCH-based therapeutics target a specific subpopulation which is a minority in the bulk of the tumor, it should not be expected that monotherapy NOTCH-targeting treatments will have a significant impact on clinical outcome. Unfortunately, many clinical trials using NOTCH therapeutics are terminated, on hold or have reported limited efficacy. Parallel to this observation, it must be noted that the current interventional setting to evaluate NOTCH inhibition efficiency is sub-optimal. The most important shortcoming of the clinical trials is the lack of robust biomarkers to select patients and monitor responses to treatment. While there are many gene expression studies investigating on- and off-target effects of NOTCH targeting using GSI, they reflect the combined effects of NOTCH1–4 receptor blockade. Since NOTCH signaling in lung tissue is complex, with redundant but also opposing functions of specific NOTCH isoforms affecting prognosis and treatment sensitivity, such signatures are unlikely to yield predictive markers for patient stratification. It is anticipated that selection of patients would improve the quality and accuracy of the information from clinical trials. Companion biomarkers that are assessed dynamically over the course of cancer treatment are needed to monitor on-target efficacy and enable therapeutic redesigning.

NOTCH receptor or ligand-specific targeting agents, as opposed to broad spectrum small molecule γ-secretase inhibitors, are more likely to induce less adverse effects, however, proper scheduling, reduced dosing, and combination with glucocorticoids can mitigate the adverse effects of GSIs. New insights into γ-secretase’s complex composition may yield receptor-specific small molecule NOTCH inhibitors (208) that could aid in attenuating the side effects of broad spectrum inhibitors. Monoclonal antibodies have minimal toxicity to the gastrointestinal tract but they generally have limited biodistribution and prolonged half-life, a shortcoming that can be addressed using F(ab′)_2_ antibody fragments which allow more flexible control over the extent and duration of inhibition. Soluble decoys of the extracellular domain of NOTCH or its ligands block receptor-ligand interaction; however, their efficacy relies on their biodistribution and pharmacokinetic properties which remain to be assessed. On the other hand, cancers may present more than one alteration that increases the activity of the NOTCH signaling pathway, therefore, the benefits of pan-NOTCH inhibitors, which target all four NOTCH receptors versus selective NOTCH receptor targeting needs further examination.

We discussed how NOTCH pathway inhibition can enhance chemo-/targeted agent sensitivity and even revert resistance in NSCLC. Although out of the scope of this review, similar opportunities exist for combining NOTCH inhibition with radiation therapy (174, 175), and potentially, immune therapies. Because NOTCH signaling impacts several features of the tumor microenvironment such as tumor hypoxia and tumor cell metabolism, the prospect of altering the microenvironment using NOTCH inhibitors is exciting since normal cells usually do not develop drug resistance, but challenging because of the adverse effects of NOTCH inhibitors on normal tissue. Recently, there has been a report that T-ALL cells restore NOTCH signaling activation upon GSI withdrawal, suggesting that the persisting cells are reversibly resistant to GSI through epigenetic alterations. Combination therapies with epigenetic modulators, however, enhance NOTCH-inhibition therapy (209). Nevertheless, it remains to be established whether epigenetic modifiers can prolong GSI effects in lung cancer with tolerable normal tissue effects.

In summary, lung cancer is a complex heterogenous disease with interpatient, intratumor and inter-/intrametastatic heterogeneity at the subtype level, therefore, successful treatment options are likely to arise from personalized precision treatment. There is paramount preclinical evidence for potent antitumor activity of NOTCH therapeutics in NSCLC but our biological understanding of the tissue and context-specific roles of NOTCH is understudied. Biomarkers will be essential to advance into clinical development to obtain meaningful and reliable answers on therapeutic ratios. Finally, while there is much attention into the development of smarter drugs to target specific drivers of progression and treatment resistance, efforts should also be directed toward identifying synergistic interactions of NOTCH inhibitors with clinically approved systemic treatments as for such combinations are likely to lead to faster clinical implementation and hence, benefit the patients.

## Literature Search Criteria

Studies including chemotherapeutics or targeted agents used as part of standard of care treatment against NSCLC, studies that included NOTCH signaling, non-squamous NSCLC, preclinical and clinical studies, and studies using GSI, monoclonal antibodies, or natural agents to target NOTCH pathway members. Whenever possible, we analyzed data from studies after the year 2000.

## Author Contributions

VSI, LG, and MV wrote the review. JT and LD assisted in the writing. VSI prepared the figure and the tables. LG prepared Table 6. MV, JT, LD supervised the study.

## Conflict of Interest Statement

The authors declare that the research was conducted in the absence of any commercial or financial relationships that could be construed as a potential conflict of interest.
